# Silica optical fiber integrated with two-dimensional materials: towards opto-electro-mechanical technology

**DOI:** 10.1038/s41377-021-00520-x

**Published:** 2021-04-14

**Authors:** Jin-hui Chen, Yi-feng Xiong, Fei Xu, Yan-qing Lu

**Affiliations:** 1 Institute of Electromagnetics and Acoustics, Xiamen University, Xiamen, 361005 China; 2 College of Engineering and Applied Sciences, National Laboratory of Solid State Microstructures and Collaborative Innovation Center of Advanced Microstructures, Nanjing University, Nanjing, 210093 China

**Keywords:** Optics and photonics, Electronics, photonics and device physics

## Abstract

In recent years, the integration of graphene and related two-dimensional (2D) materials in optical fibers have stimulated significant advances in all-fiber photonics and optoelectronics. The conventional passive silica fiber devices with 2D materials are empowered for enhancing light-matter interactions and are applied for manipulating light beams in respect of their polarization, phase, intensity and frequency, and even realizing the active photo-electric conversion and electro-optic modulation, which paves a new route to the integrated multifunctional all-fiber optoelectronic system. This article reviews the fast-progress field of hybrid 2D-materials-optical-fiber for the opto-electro-mechanical devices. The challenges and opportunities in this field for future development are discussed.

## Introduction

Low-loss silica optical fibers, semiconductor lasers and erbium-doped fiber amplifiers lay the foundations of the modern optical communications. In addition to primarily transporting the lightwave, silica optical fibers have found broad applications in the distributed optical sensing^1,2^, endoscope imaging^3–5^, optical trapping^6,7^, fiber lasers^8,9^ and nonlinear optics^10^. With the development of materials science and manufacture technology, the conventional homogeneous doped core and pure cladding structures in a silica fiber have evolved with a new paradigm shift by merging the multi-structures and multi-materials. This emerging trends in optical fiber aim to break the fundamental limit by a single structure and material, and extend their photonic and optoelectronic applications.

In 1978, Hill et al.^11^ demonstrated one-dimensional fiber Bragg gratings (FBGs) employing the photosensitivity in germania-doped fiber. Later, the FBGs have found broad applications in optical communications and sensor systems^11,12^, and have stimulated many other in-fiber grating structures^13,14^. In the same year, Yeh et al.^15^ proposed the Bragg fiber in which concentric rings of alternating high- and low-refractive index are arranged, to realize lossless propagation in a core of lower refractive index than that of the cladding. From the late 20-century to the dawn of the 21-century, it witnessed booming development of optical fiber technology. Russell et al.^16,17^ successfully combined the concept of two-dimensional (2D) photonic bandgap with the fiber drawing technology, and fabricated the photonic crystal fiber (PCF), which opens a new horizon for in-fiber manipulating optical wavelength, modes, dispersions, polarizations, and nonlinearities^18^. Tong and Knight et al.^19,20^ minimized the width of waveguides and demonstrated the subwavelength silica fiber for low-loss optical waveguiding. The optical microfiber/nanofibers possess many intriguing properties^21^, such as strong field confinement, large evanescent fields and great configurability, and they have been widely used as micro- or nano-scale probes in physical, chemical, biological and materials research^21–25^. Fink et al.^26,27^ focused on integrating multi-materials with disparate electrical, optical, mechanical and thermal properties into a single fiber, with an ambitious goal of realizing multifunctional fiber devices that see, hear, sense and communicate^26,28,29^. The multimaterial fibers are an important milestone in the development of fiber devices, while it is challenging to seamlessly connect with the universal silica optical fiber networks due to the mode-field mismatch and fiber splicing difficulties.

**Fig. 1:**
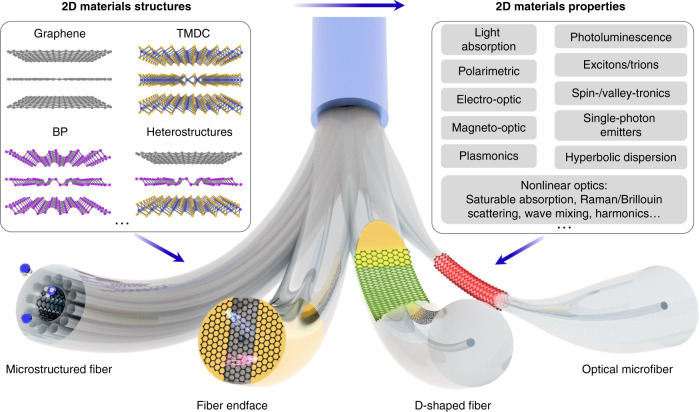
Schematic of 2D materials integration to optical fiber platform. Left box illustrates the crystal structures of multilayered graphene, transition metal dichalcogenides (TMDC), black phosphorus (BP) and their heterojunctions. Right box lists typical optical and optoelectronic properties of 2D materials. 2D materials are flexibly assembled in microstructured fiber, fiber endface, D-shaped fiber and optical microfiber for versatile applications

During this period, the Dirac fermionic graphene in condensed matter physics was emerging rapidly since the seminal work of Geim and Novoselov et al.^30,31^, and it demonstrated many supreme properties such as carrier mobility, thermal conductivity, light absorption, mechanical stiffness/strength and chemical functionalization^32–34^. The rise of graphene and related 2D materials has brought profound impact on nearly every field related to electronics, photonics, chemistry, energy and biology^32–37^. With benefit of hindsight, the 2D materials with salient optoelectronic and mechanical properties are fully incorporated complementarily to the passive silica optical fibers benefiting from their flexibility, configurability and versatility, as shown in Fig. 1. The main advances enabled by 2D materials are post-processing on the conventional passive silica fiber structures to realize light emission, modulation, switching and detection^24,32,38–43^, which paves the way to all-fiber multifunction-integrated optoelectronics^26,29,38^. The pioneering work of the fiber-integration-2D-materials, such as ultrafast fiber laser^44,45^, graphene polarizer^46,47^, fiber-optic sensor^48–54^ and all-optical modulator^55–58^ have been experimentally realized. Although there is a plethora of comprehensive review papers on 2D materials optoelectronics^32,36,38–40,59^, none of them provide the full pictures and prospects of optical fiber integration. Therefore, we aim to review the fast-growing research field of hybrid fiber-2D-materials for the opto-electro-mechanical technology. In this article, we first summarize the basic properties of fused silica and three typical 2D materials i.e., graphene, transition metal dichalcogenides (TMDC), and black phosphorus (BP), and particular emphasis is put on the tunability of their linear and nonlinear optical properties. Next, we analyze four kinds of fiber structures integrated with 2D materials (Fig. 1), each of which has their own uniqueness. Then we discuss the all-fiber photonic and optoelectronic applications, i.e. fiber polarizers, light emitting devices, optical modulators, photodetectors, optical sensors and nonlinear optics. Finally, we discuss the challenges and opportunities in the optical-fiber-2D-materials towards the practical applications, and provide our vision for the future perspectives in this field.

## Silica and 2D materials properties

### Silica

**Fig. 2:**
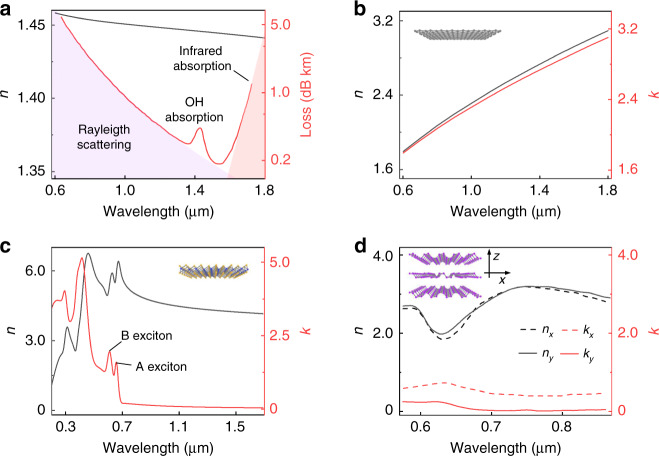
Optical dispersions of fused silica and 2D materials. **a** Refractive index *n* and optical loss (log plot) of silica glass^62^. **b** Refractive index *n* and extinction coefficient *k* of monolayer graphene with the following calculated parameters: Fermi level 0.1 eV, scattering time 100 fs, temperature 298 K, graphene thickness 0.34 nm. Optical dispersion (*n*, *k*) of monolayer MoS_2_ (**c**)^115^ and thick BP film with strong anisotropy (**d**)^162^. The thickness of monolayer MoS_2_ is set as 0.71 nm. **a** Reproduced from ref. ^62^, with the permission of Springer Nature Switzerland AG 2019. **c** Reproduced from ref. ^115^, with the permission of AIP Publishing. **d** Reproduced from ref. ^162^, with the permission of OSA Publishing

Commercialized standard optical fiber is made of fused silica for their intrinsic ultralow optical loss in the visible and near infrared band (Fig. 2a). The first low-loss optical fiber was invented in 1970 at the Corning thanks to the seminal work of Kao and Hockham et al.^60^ who raised the idea that the attenuation in optical fibers caused by impurities could be removed. In current optical fibers, three main loss contributions still exist, i.e., Rayleigh scattering at short wavelength due to the inhomogeneous glass, the infrared lattice vibration and the residual OH absorption, and the state-of-the-art optical fiber demonstrates propagating loss of ~0.15 dB/km^61,62^. Under the regime of linear optics, the isotropic silica has a moderate refractive index of ~1.45, and the dispersion (Fig. 2a) is well approximated by the Sellmeier formula, which is widely used for characterizing optical materials,

1 $$n(\lambda ) = \sqrt {1 + \mathop {\sum}\limits_j {\frac{{a_j\lambda ^2}}{{\lambda ^2 - b_j}}} }$$

where *λ* is the light wavelength, *a*_*j*_ and *b*_*j*_ are Sellmeier coefficients. As for the nonlinear optics, the primary term of isotropic silica is third-order nonlinearity (*χ*^(3)^) with the moderate value of ~10^−20^ m^2^/W. Due to the ultralong light-matter interaction in silica fiber, efficient nonlinear optics have been revealed, such as Kerr effect, four-wave mixing, stimulated Raman and Brillouin scattering^10^. While under the electric-dipolar approximation the bulk second-order nonlinearity (*χ*^(2)^) in fused silica is missing, researches show that the structural loss of inversion symmetry at the surface/interface allows second harmonic generation (SHG) with the surface *χ*^(2)^ of the order ~10^−21^ m^2^/V^63^. Further, the electric-quadrupole and magnetic-dipole response also contribute to the SHG response^64^. As for mechanics, the Young’s modulus of bulk silica glass is approximately 70 GPa. Nevertheless, the measured strength is as low as 0.2 GPa because of the surface imperfections^65^. Intriguingly, Brambilla et al.^65^ recorded the maximum strength of ~26 GPa in silica nanowires with significantly reduced defects.

### 2D materials

2D materials are a class of crystals whose thickness vary from one-atomic layer to tens of nanometers, and most of them are formed by in-plane covalent bonds and out-of-plane Van der Waals force^66^. The simultaneously high stiffness and elasticity/flexibility in 2D materials enables their adaptation into various photonic structures, enhancing the light-matter interactions. Since the first discovery of graphene, the burgeoning development of materials synthesis has significantly expanded the library of 2D material from the elements to compounds^67–70^, and more than 600 stable 2D layered materials are predicted^71^. In contrast, only a few mainstay 2D materials are successfully integrated to the optical fiber platform, such as graphene, TMDCs and BP. These 2D materials spans the electronic bandgap of 0–2 eV, which corresponds to the optical spectral response from the terahertz to visible band, and is sufficient for optical fiber photonics and optoelectronics. There is still huge potential to discover in fiber integration with other novel 2D materials.

### Graphene

As the first discovered 2D materials, the atomic-thin graphene with honeycomb structures has been extensively studied for more than 15 years^32–36,38^, and it still shows strong vitality especially in condensed matter physics so far, for example, the magic-twist graphene layers for superconductor and correlated insulator^72,73^. Conceptually, graphene can be considered as a mother material for fullerene, nanotubes and graphite^74^, while the dimensionality defines their drastic difference. Graphene has an ambipolar electric field effect with carrier mobility reach 10^6^ cm^2^ V^−1^ s^−1^ due to its massless Dirac fermions^75^, which is 2-3 orders of magnitude higher than that of the semiconductor silicon, and have found applications in high-frequency transistors^76,77^. The high electron velocity and linear energy-momentum dispersion contribute to graphene’s intriguing optical properties, for example broadband light absorption (Fig. 2b). Generally, the linear optical response of graphene can be determined by a surface optical conductivity from Kubo formula, and under the assumption ${K_B}T < < \vert {\mu_c} \vert$, the optical conductivity can be analytically derived as^78,79^

2 $$\sigma (\omega ) = - \frac{{ie^2\mu _c}}{{\pi \hbar ^2(\omega \, - \, i\tau ^{ - 1})}} - \frac{{ie^2}}{{4\pi \hbar }}{\mathrm{ln}}\left( {\frac{{2\left| {\mu _c} \right| - (\omega \, - \, i\tau ^{ - 1})\hbar }}{{2\left| {\mu _c} \right| + (\omega \, - \, i\tau ^{ - 1})\hbar }}} \right)$$

where *k*_*B*_ is the Boltzmann’s constant, *e* is the electron charge, ℏ is the reduced Planck’s constant, *τ* is the relaxation time. The first and second part of Eq. (2) are contributed by the graphene intraband and interband transition, respectively. In the visible and near-infrared spectra with ℏ*ω* > 2|*μ*_*c*_ | , the interband transition dominates, and the graphene’s optical conductivity is ~*e*^2^/4ℏ, which directly determines the universal light absorption of ~2.3% per layer^80^. Moreover, the light absorption can be simply tuned by the electric, optical and magnetic field^81–84^, strain gauge^85,86^, and even the molecule adsorption^87,88^. Since the second-order nonlinearity is forbidden in centrosymmetric graphene under the electric-dipole approximation, third-order nonlinearity is the dominant effect with *χ*^(3)^ ~ 10^−17^ m^2^/W^44,45,89–91^. In particular, the broadband, low-threshold-power and ultrafast-response saturable absorption of graphene has attracted great research interest in pulsed fiber laser and all-optical modulation^42,43,92,93^. Manipulating the nonlinear optical absorption is also realized by engineering the Fermi-Dirac distribution^94–96^. Recent experiments systematically reveal the tunable enhancement of third harmonic and four-wave mixing by the Dirac conical bandstructure^97,98^. Note that there is relentless effort in opening graphene’s second-order nonlinearity through symmetry breaking, for example, the electric field induced nonlinear effects^99^. High harmonic generation in graphene is observed and enhanced by elliptically polarized light excitation, and this finding sheds light on the possibility of strong field and ultrafast nonlinear dynamics in massless Dirac fermionic materials^100,101^.

The in-plane strong covalent bonds in graphene determine its thermal stability and mechanical strength. Thermally, monolayer graphene is stable in oxygen atmosphere withstanding high temperature of ~300 °C, and the oxidation temperature is up to 500 °C for multilayers^102^. Mechanically, suspended defect-free graphene shows Young’s modulus of ~1.0 TPa and intrinsic strength of 130 GPa^103^, and is highly flexible with a failure strain up to 11%^104^. The supreme mechanical properties of graphene enable the excellent conformal coating to the optical fiber system.

### TMDCs

TMDCs have a large group of materials with the formula MX_2_, where M is a transition metal element from group IV-VI (such as Mo, W, Ti, Nb, Zr) and X is a chalcogen (such as S, Se, Te), and there are many comprehensive reviews on TMDCs^59,105–107^. Here we focus on the most studied MoS_2_ (or WS_2_) for its robustness in monolayer limit at room temperature, which benefits for practical optoelectronic devices. Layered MoS_2_ evolves a transition from indirect bandgap (bulk, 1.2 eV) to direct bandgap (monolayer, 1.9 eV) semiconductor due to the lateral quantum confinement effect, and the measured quantum yield of photoluminescence in monolayer crystal is 10^4^ higher than that of the bulk crystal^108,109^. The enhanced Coulomb interaction due to the low-dimensional effects in TMDCs forms the tightly bound excitons and trions^110–112^, and they are tunable by electric field and strain gauge^113,114^. Thus, the optical dielectric function of monolayer MoS_2_ is strongly correlated to the exciton energy^115,116^ in the visible spectra, as shown in Fig. 2c. Moreover, the spin-orbit coupling together with the time-reversal symmetry in monolayer MoS_2_ leads to valley-contrasting optical dichroism^117–119^, which demonstrates the viability of optical valley control and valleytronics, and finds applications in photonic crystals, plasmonics and waveguides^120–124^. For nonlinear optics, the measured surface second-order nonlinearity in monolayer MoS_2_ is on the order of 10^−17^–10^−19^ m^2^/V^125–127^, and the large discrepancy is probably due to the experiment configurations and sample qualities. Researches show that the *χ*^(2)^ nonlinear optics in MoS_2_ is highly dependent on the layer number^125–127^, stacking order^128,129^, pump wavelength^126,130^, edge state^131^, and even the electrostatic doping^132^. The third-harmonic nonlinear susceptibilities of MoS_2_ is comparable to that of conventional semiconductors under resonant conditions (~10^-17^ m^2^/W)^133^. It is revealed that the few-layer MoS_2_ exhibits significant saturable absorption effects^134,135^, and that monolayer has a strong two photon absorption coefficient as high as 7.6 × 10^−8^ m/W, which is three orders of magnitude larger than that of conventional semiconductors^136^.

Mechanically, suspended monolayer MoS_2_ exhibits Young’s modulus of 270 GPa and intrinsic strength of ~23 GPa^137^. While the 2D TMDCs can exist in multiple crystal structures with distinct electrical properties, all Mo- and W-based TMDCs except WTe_2_ are stable in trigonal prismatic phase (hexagonal symmetry) under ambient conditions^105,138^. In addition, the strong electromechanical coupling in TMDCs have enabled structural phase switching by a variety of stimuli, such as chemical doping^139^, mechanical deformation^140,141^ and electrostatic gating^142,143^. The dynamic control of structural phase transition in TMDCs may find applications in phase-change electronic and photonic devices. Recently, the valley-mechanical coupling in monolayer MoS_2_ is experimentally realized, and it is controlled by pump light, magnetic field gradient and temperature, which paves the way to valley-actuated devices and hybrid valley quantum systems^144^.

### Black phosphorus

Black phosphorus attracts regenerated interest as anisotropic layered materials for electronics and optoelectronics, since it fills the energy gap between semi-metallic graphene and semiconducting TMDCs (1-2 eV) with high carrier mobility^145–148^, which is suitable for infrared optoelectronics. Due to the interlayer coupling, the bandgap of BP highly depends on the layer numbers from 0.3 eV (bulk) to 1.7 eV (monolayer) with a power law *E*_opt_ = 1.486/*N*^0.686^ + 0.295, where *E*_opt_ is the optical gap in unit of eV, and *N* is the layer number^149–153^. Intriguingly, BP always exhibits direct bandgap for various layers, and it is promising for efficient infrared light detection^154–156^ and emission^149–151,157^. The puckered crystal structure endows BP with strong electronic, photonic and mechanical anisotropy^145,150,153,154,158,159^ in contrast to graphene and TMDCs, as shown in Fig. 2d. It is revealed that conductivity along the armchair direction is much higher than along the zigzag direction^145,160^, and the excitons (binding energy 0.3-0.9 eV) and trions (binding energy ~ 0.1 eV) in monolayer BP are also highly anisotropic and robust^149,161^. The linear dichroism in BP (Fig. 2d) can be indicated from the optical selection rule^154,162^, thus their crystalline direction is easily determined through polarization-resolved spectroscopy^145,151,154^. For the nonlinear optics, the third-order nonlinearity of BP is comparable to graphene and TMDCs, and saturable absorption^163,164^, four-wave mixing^165,166^ and third harmonic generation^167,168^ are observed in BP film.

Regarding mechanics, the theoretical in-plane Young’s modulus is 41.3 GPa (106.4 GPa) along the armchair (zigzag) direction in BP, and the sustained strain can be as high as 0.48 (0.11) along armchair (zigzag) direction owing to the puckered configuration^158^. Tao et al.^169^ measured the Young’s modulus of few-layer BP averagely to be 27.2 GPa and 58.6 GPa in armchair and zigzag directions, respectively. The strain effect is extensively researched for anisotropic modulating the electrical and optical functions of BP^170–173^. Although the bulk BP is the most stable phosphorus allotrope at room temperature, the few-layer BP is vulnerable to oxygen and water, which hinders their practical applications. In the past years, the stability of BP is comprehensively studied, and many effective passivation techniques are developed, such as the surface encapsulation with Al_2_O_3_, SiO_2_ and graphene, and the structural modifications^174^.

### 2D materials fiber integration

The integrations of 2D materials to the optical fibers have various architectures based on different materials transfer processes^39,88,175^. According to the light-matter interaction length, the 2D-materials-fiber structures can be categorized into two groups: fiber-endface and guided-waveguide integration as shown in Fig. 1. The cleaved optical fiber endface is an intriguing platform since it maintains nearly free-space light coupling and manipulation along with remote and self-aligned optical path. Graphene integration to fiber endface is firstly explored for saturable absorption in ultrafast fiber laser for its easy fabrications^44,45^, while suffering from the short light-graphene interaction length and poor heat dissipation. Researchers usually employed 2D-materials-polymer composites and sandwiched them between two fiber connectors^45^. With shrinking light-based technology, such as plasmonics, photonic crystal and metamaterials/metasurface, the multi-structures and materials on the fiber endface will promise novel optical fiber optoelectronics^176,177^. When 2D materials integrated on a fiber capillary tip, the unique free-standing diaphragm of atomic thickness enables ultrasensitive all-fiber microelectromechanical system (MEMS)^50,51,178^.

**Fig. 3:**
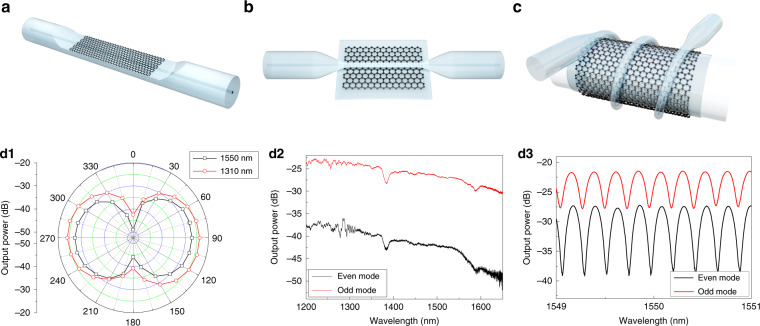
Polarimetric fiber devices with graphene. **a** D-shaped fiber laminated with graphene. **b** Optical microfiber (MF) on graphene-substrate. **c** Stereo MF-graphene structure with adjacent coil coupled or isolated. Fiber polarizer characterizations in stereo MF-graphene devices by two-coil structure (**d1**–**d2**)^47^. **d3** Transmission spectra of the MF-graphene coil-resonator for two orthogonal modes^47^. **d1**–**d3** Reproduced from ref. ^47^ with the permission of OSA Publishing

Waveguide integration means that light-matter interaction strength depends on the geometry scale of 2D materials along the wave propagation, which is free from their atomic-thickness limit. There are mainly three waveguide coupling architectures, i.e. D-shaped fiber (DSF), optical microfiber (MF) and microstructured fiber. In particular, DSF is fabricated either by side-polishing or chemical etching to expose the fiber core and enhance the surface evanescent field^179^, and the flat surface structure is beneficial for excellent contact with grafted 2D materials^46,96,180,181^ as illustrated in Fig. 3a. While less explored, the DSF embedded with nanophotonic structures and functionalized by advanced materials are of great potentials for all-fiber light-manipulation with robustness^176,179^. As for MF integration, the 2D materials, for example graphene, is either wrapped around or line-contacted with an MF (Fig. 1 and Fig. 3b, c). Generally, an MF is continuously tapered or chemically etched from a standard optical fiber (~125 μm), the diameter of which ranges from hundreds of nanometers to tens of micrometers^21,23,24^. For subwavelength MF, the large evanescent field enables strong interactions with 2D materials^56,182^, and experimental results show that hybrid MF-WS_2_ of sub-100 μm interaction length is sufficient for >95% light absorption^182^. Technically, the surface encapsulated MFs of sub-micrometer diameters are difficult to fabricate and handle, because they are easily broken or contaminated in ambient environment. Xu et al. reported a robust stereo MF-graphene structure with lab-on-a-rod technique^47,52,58,183^. Since the fabrication process only involves laminating a small piece of graphene onto a rod of millimeters diameter, and the MF is helically winded around a surface functionalized rod, arbitrary light-graphene interaction length can be realized with miniaturized size, as shown in Fig. 3c. Another uniqueness is the formation of optical resonators through inter-coupling between adjacent coils for cavity-enhanced interactions^47^. Note that most of the researches focus on the light absorption or spectral shift functions by the fiber-2D-material devices^47,55–57,88,184–186^, the exceptional valleytronics^117–119^, excitonics^112,161^, single photo-emitters^187,188^ and optical-nonlinearity^189,190^ in 2D materials interact with chiral field of MF^191^ deserves in-depth explorations, which would stimulate all-fiber applications for nanolasers^192^, chiral photonics^122^ and quantum optics^193^.

The microstructured fibers with ingenious microfluidic channels such as photonic crystal fiber (PCF) and hollow core fiber (HCF) are another intriguing platform to accommodate various materials in the hole walls. Indeed, PCF infiltrated with gas, liquids, glasses, semiconductors and metals have significantly extended their functionality in the linear and nonlinear optics^194^. Integrating 2D materials in PCF is nontrivial since the 2D materials of atomic thickness not only keep PCF structure and optical functions intact, but also they will perform unique functions that cannot be realized in conventional materials^195,196^. As for the device preparations, solution processed 2D materials are infiltrated into the air hole of PCF or HCF using a pump, and a thin film is deposited after solvent evaporation^197,198^. The solution injection technique is simple, while the quality of infiltrated film is poor, which significantly limits their further applications. Recently, Liu et al.^195,199^ reported a direct chemical vapor deposition (CVD) growth method, and realized massive production of graphene-PCF/HCF with high crystalline quality and environmental adaptability. The breakthrough-work opens new possibilities for scientific research and practical applications in all-fiber optoelectronics.

### Photonic and optoelectronic fiber devices

Motivated by the intriguing physical properties of graphene and related 2D materials, many on-chip monolithic photonic and electronic devices are created and developed in the past years^36,38,39,41–43,59^. In contrast, the development of all-fiber photonics and optoelectronics with 2D materials shows much slower pace. This section reviews the mainstream applications of 2D-materials-optical-fiber in the categories of polarizers, light-emitting devices, optical modulators, photo-detectors, optical sensors and nonlinear optics.

### Fiber polarizers

Optical fiber polarizers operate in-line discriminating polarized light transmission with high extinction ratio, which is important in communication, sensor and laser systems. The conventional in-fiber polarizers are based either on asymmetrically polarization-dependent coupling with external materials such as birefringent dielectrics and plasmonic metals, or on single polarization fiber^200^. The graphene-based fiber polarizers have flexible structure designs and tunable functions by electric-gating (Fermi level)^46,96,201^, and there are trade-offs between insertion loss and polarization extinction ratio. Bao et al.^46^, demonstrated ultra-broadband (visible to infrared), high extinction ratio (27 dB) fiber polarizers on a DSF-graphene structure (Fig. 3a). It is figured that high-order leaky modes with transverse magnetic (TM) polarization suffers larger loss in graphene than with transverse electric polarization (TE), which contributes to the TE-pass polarizer^46^. Later works show that under guided-mode interaction scheme, the pass-polarization can be either TE or TM on various waveguide structures^47,96,180,202^, and is fundamentally determined by the in-plane electric field distribution in graphene layers^201,203^. Kou et al.^47^ tailored the stereo graphene-MF structure, and they realized a high extinction ratio fiber polarizer (~16 dB @ 1550 nm, Fig. 3d1–d2) and high-*Q* (2 × 10^4^) single-polarization fiber resonator (Fig. 3d3) by controlling the near-field coupling between adjacent MF coils. Note that when the adjacent MF coil is decoupled, the stereo-MF-graphene structure is physically equivalent to MF-on-graphene as illustrated in Fig. 3b; since the geometry scale of functionalized rod (~ mm) is far larger than MF (~ μm), the spatial curvature and Berry phase can be neglected. Besides graphene and its derivatives, the strong anisotropic 2D materials such as BP^145,154^ and ReS_2_^204,205^ are promising candidates for polarimetric fiber components.

### Light-emitting devices

**Fig. 4:**
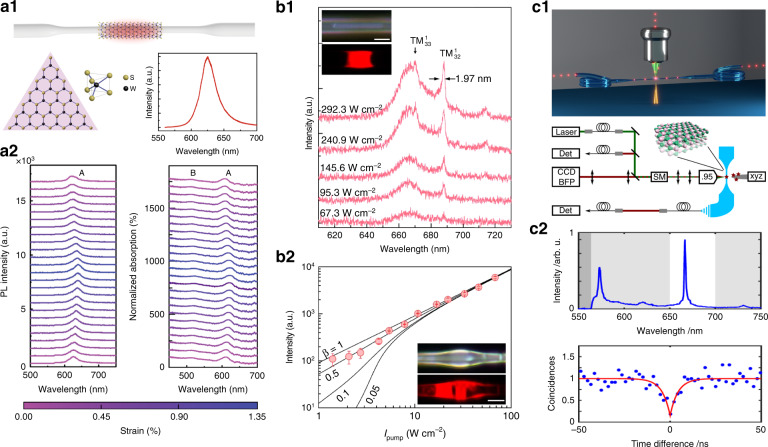
Light emitters integrated in optical microfiber. **a1** Schematic hybrid MF-WS_2_ structure for enhanced light emission^182^. Inset: Crystal structure of a triangular monocrystalline WS_2_ (left panel), and photoluminescence (PL) spectra of a monolayer WS_2_ (right panel)^182^. **a2** In-line strain manipulation of the PL (left panel) and absorption spectra (right panel) of hybrid MF-WS_2_^182^. Room-temperature continuous wave lasing from as-photoactivated monolayer MoS_2_ in MF resonator (**b1**) and microbottle resonator (**b2**)^192^. **c1** Coupling quantum emitters of hBN with MF waveguide^211^. **c2** Spectra of the light collected through the fiber (top), and anti-bunching measurements of the light collected through the fiber (bottom)^211^. **a1**–**a2** From ref. ^182^. Reproduced by permission from Springer Nature: Light: Science & Applications. **b1**–**b2** Reprinted by permission from AAAS^192^. **c1**–**c2** From ref. ^211^. Reprinted by permission from ACS Publications

In principle, 2D materials with direct electronic bandgap are potentially efficient light emitters in the process of excited electrons recombination with holes, and layered TMDCs^108,109,112,206^, BP^149,150^ and their heterostructures^207^ are extensively studied for on-chip light emitting devices. Nevertheless, the high-performance fiber-emitting devices are much less researched. Chen et al.^182^ reported monolayer monocrystalline WS_2_ transferred to silica MFs, and they observed tunable and strong excitonic photoluminescence (PL) by strain gauge (Fig. 4a1–a2), in contrast to the background spectra from defects and doping in the fiber itself. The enhanced PL are contributed by the near-field light interaction and collection (efficiency ~12%). Recently, Liao et al.^192^ used a simple photoactivation method to improve the room-temperature quantum yields of monolayer MoS_2_ directly grown onto silica microfibers, by more than two orders of magnitude in a wide pump power range, which allows direct lasing with strikingly reduced thresholds down to 5 W/cm^2^ (Fig. 4b1–b2). In addition to the classical light generation, the nontrivial single photon emitters (SPEs) in defect or strained 2D material are attracting attentions^187,188,208,209^, and the in-fiber SPEs offer alignment-free collections and near-resonant excitation schemes. The ideal on-demand SPE emits exactly one photon at a time into a given spatiotemporal mode, and all photons are indistinguishable^210^. Schell et al.^211^ demonstrated coupling of SPEs from 2D hexagonal boron nitride to a tapered MF (Fig. 4c1–c2), and found a collection efficiency of 10% in the system. The performance of SPEs can be significantly improved by fiber-cavity structures^212^. Exploring 2D materials that generating telecom band (~1550 nm) SPEs is strongly required in the optical-fiber quantum networks^209^. The ultimate goals of on demand, highly pure, and coherent SPEs integrated with optical fiber remains to be solved.

### Optical modulators

**Fig. 5:**
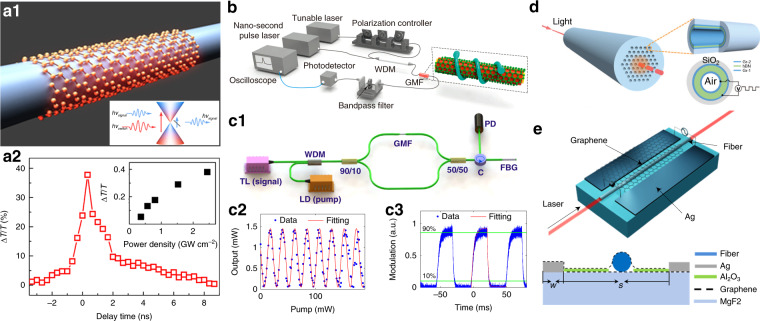
Optical fiber modulators integrated with 2D materials. **a1** Schematic illustration of a graphene-coated microfiber (GMF) structure for all-optical modulator. Inset: Schematic describes Pauli blocking effect^56^. **a2** Pump−probe delay time measurement results. Inset: Relation of the modulation depth and pump intensity^56^. **b** Measurement setup of the pump-probe system with a stereo graphene-MF device^58^. Experimental setup for measuring the phase shift in GMF (**c1**), all-optical switching by a GMF (**c2**), and temporal response of the all-optical switching (**c3**)^57^. **d** Schematic sketch of a sandwiched graphene/hBN/graphene photonic-crystal-fiber electro-optic modulator (EOM)^229^. **e** Schematic diagram of the MF-based EOM (upper panel) and its cross-section structure (lower panel)^227^. **a1**–**a2** Reproduced from ref. ^56^ with permission of ACS publications. **b** Reproduced from ref. ^58^ with permission of Springer Nature: Light: Science & Applications. **c1**–**c3** From ref. ^57^. Reprinted by permission of OSA Publishing. **d** Reproduced from ref. ^229^ with permission of The Royal Society of Chemistry. **e** Reprinted from ref. ^227^ by the permission of IEEE Publishing

Optical modulators are the essential components in photonics and optoelectronics, which operate at encoding information into the light beams. 2D materials with supreme and tunable photo-response functions by external fields, have driven significant advances of optical modulators, and there are several comprehensive reviews in this topics^42,43,213^. Here we focus on the fiber-compatible modulators. The all-optical modulator (AOM) that uses one light beam to control the transmission of another one, can realize ultrafast modulation speed avoiding the electrical bottleneck. In principle, the AOM based on Pauli-blocking effect^55,56,58,180,214,215^, Kerr effect^216,217^ and opto-thermal effect^57,218–221^ are widely studied in various 2D materials such as graphene, BP and TMDC^42,43,213,222–224^. Liu et al.^55^ first reported broadband all-optical modulation using a graphene-covered-microfiber (GMF) structure. Later, Li et al.^56^ pushed the response-time of GMF to the carrier-relaxation limit of graphene ~2.2 ps though with a small modulation depth (MD) of ~1.4 dB, as shown in Fig. 5a1–a2. It is challenging to fabricate and manipulate such sub-wavelength GMF (~1 μm) for practical applications. Chen et al.^58^ realized a robust stereo GMF structure (Fig. 5b) for polarization-dependent light modulation with a maximized MD of ~7.5 dB and a modulation efficiency of ~ 0.2 dB/mW. Gan et al.^57^ demonstrated an all-fiber phase shifter assisted by graphene’s photothermal effect, and they obtained a phase shift exceeding 21π with a maximized slope of 0.192 π/mW, as shown in Fig. 5c1–c3. Towards the practical applications, the performance indexes of fiber AOM such as the control power consumption, switching time, MD and insertion loss need to be globally optimized and balanced^225^. For example, the larger MD generally requires enhanced light-matter interactions either through field confinement or interaction length, which often brings higher insertion loss from materials absorption and scattering; the geometry scale of device, such as waveguide diameter and length may also influence the ultimate switching time^42^.

The electro-optic modulators (EOM) implementing electric field to control the light properties are particularly desirable in current communication networks. Although there have been substantial achievements for on-chip EOM with graphene^42,43,213,226^, based on the tunable electro-absorption or electro-refractive effects. It is nontrivial to develop high performance all-fiber EOM^227,228^ for their seamless connection to the mainstay optical fiber systems. Xu et al.^227^ proposed a high-speed traveling-wave EOM on a graphene/MF structure with a 3 dB bandwidth of 82 GHz, as shown in Fig. 5e. Experimentally, Lee et al.^96^ demonstrated ion liquid gating (~3 V) in multilayered-graphene-DSF with MD of ~10 dB for TE polarization. Liu et al.^195^ reported a graphene-PCF EOM with large MD of ~ 20 dB/cm under ~2 V gate voltage. These work use ion liquid as efficient gating medium while suffering low modulation speed and long-term stability. For high-speed modulations, the adaptation of solid gating-dielectric (Fig. 5d–e), sophisticated circuit design and 2D-materials engineering in fiber EOM are worth of further research^226,229,230^.

Besides the aforementioned modulating configurations, the acoustic-optic, mageto-optic, elastic-optic, electro-mechanical and valley-optomechanical effects in 2D materials are potential candidates for optical modulators^42,43,144,231^. For example, the unique elastic-optic response in graphene has enabled mechanical intensity-modulation in the GMF structure with an MD of ~0.04 dB/mm under 1% strain (MF diameter of 5 μm), and the modulation rate can reach hundreds of kilohertz^184^.

### Photo-detectors

**Fig. 6:**
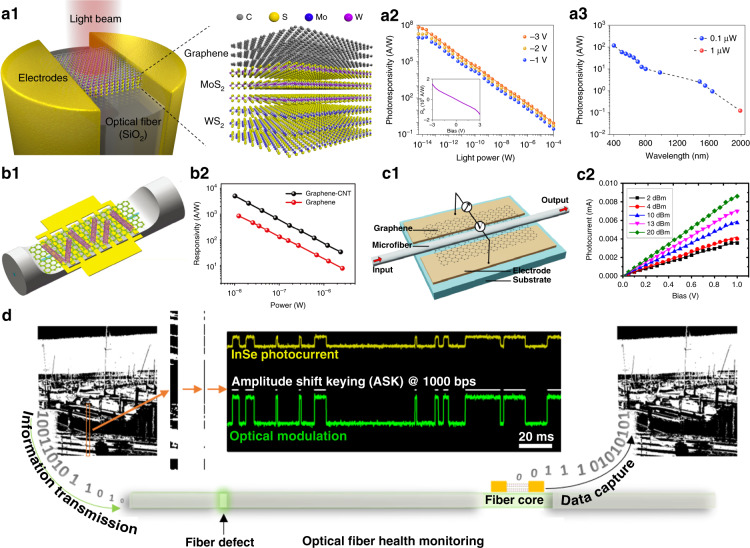
State-of-the-art all-fiber photodetectors (FPD) with 2D materials. FPD based on fiber-endface integrated graphene-MoS_2_-WS_2_ heterostructures (**a1**) for ultrasensitive photodetection (**a2**) and broadband spectra response (**a3**)^236^. Schematic of FPD fabricated in DSF with carbon nanotube-graphene heterojunction and interdigitated metal electrodes (**b1**) for enhanced photodetection (**b2**)^239^. Schematic of FPD by the microfiber-on-graphene structure (**c1**) and its photocurrent generation with bias voltage (**c2**)^234^. **d** Binary image transmitted in an optical fiber and near-field detected by an InSe FPD deposited on the surface of the multimode fiber^240^. **a1**–**a3** Reprinted from ref. ^236^. Copyright 2018, Wiley-VCH. **b1**–**b2** From ref. ^239^ by the permission of The Royal Society of Chemistry. **c1**–**c2** Reproduced from ref. ^234^ with the permission of OSA Publishing. **d** From ref. ^240^. Reprinted by permission from ACS Publications

Photodetectors convert light signals into electrical signals that can be processed by standard electronic circuits. Conventionally, the in-fiber optical signals are out-coupled and detected by external planar photodetectors, which are fabricated on silicon or other bulk semiconductors. The development of 2D materials brings new possibilities to realize all-fiber photodetectors (FPD), since they are of broad photo-response spectra and highly mechanical flexibility without any need of epitaxial substrate^232,233^. Sun et al.^234^ demonstrated broadband (1500 nm–1600 nm) photodetection in a microfiber-graphene photoconductive device, while the photocurrent responsivity is as small as ~2.81 mA/W as shown in Fig. 6c1–c2. Chen et al.^235^ fabricated visible-light response FPD by directly bonding few-layer MoS_2_ to a fiber endface along with paired gold electrodes. Furthermore, they employed Van der Waals heterostructures to improve device performances^236–238^. For example, using multilayer graphene-MoS_2_-WS_2_ with layer-by-layer transfer method, an ultrahigh responsivity of 6.6 × 10^7^ A/W (Fig. 6a1–a2) and a time response of ~7 ms at 400 nm light wavelength were achieved. The sub-band transitions and photogating effect in the heterostructures enable broadband spectra detection ranging from 400–2000 nm with high responsivity (Fig. 6a3)^236^. Recently, Zhuo et al.^239^ assembled a hybrid carbon nanotubes/graphene on a DSF, and they realized a maximized photoresponsivity of ~1.48 × 10^5^ A/W (Fig. 6b1–b2)^239^. Jin et al.^240^ developed a clean device transfer technique and realize near-field coupled 2D InSe photodetectors on surface of a multimode fiber with fast response time (~67 μs). A proof-of-concept binary image transmittance and detection by the InSe FPD was demonstrated, as shown in Fig. 6d. Note that given the figures-of-merit in photodetectors^232,233,241^, i.e. responsivity, electrical/optical bandwidth and noise equivalent power, there are still many technical issues to be solved in FPD devices compared with the on-chip photodetecting architectures^232,241^, since the sophisticated micro-/nano-fabrication technology and diversified 2D materials transfer/processing are stringently lacking in current optical fiber platform. Extending 2D library, combining nanophotonic structures and advancing electrical designs in fiber-endface and DSF platforms are exciting areas to be explored^176,230,232,233,239,241–243^.

### Optical sensors

**Fig. 7:**
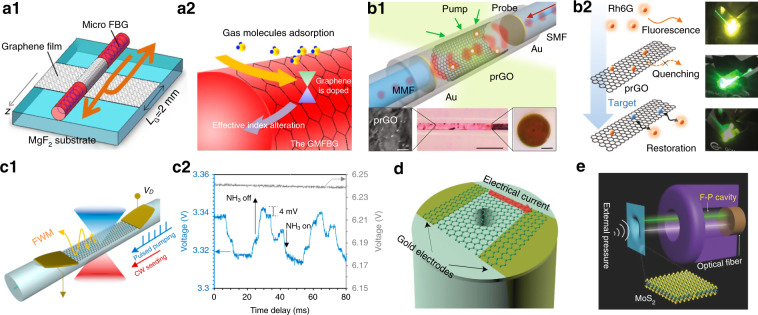
Optical fiber sensors with 2D materials. Structure of graphene-coated microfiber-based Bragg grating (**a1**) and its molecules sensing principle (**a2**)^48^. **b1** Photonic biosensor by depositing partially reduced graphene oxide (prGO) on a microfluidic dye resonator. Inset: Microscopic pictures of the devices^53^. **b2** Fluorescent resonance energy transfer sensing mechanism^53^. Electrically tunable four-wave-mixing in graphene-D-shaped-fiber (**c1**) for individual gas molecule (NH_3_) on/off dynamics detection (**c2**)^54^. **d** Miniature optical fiber current sensor based on a free-standing graphene with two gold electrodes on the pre-etched fiber tip^51^. **e** Schematic illustration of the F-P pressure sensor based on few-layer MoS_2_ and its working mechanism^178^. **a1**–**a2** Reproduced from ref. ^48^ with the permission of OSA Publishing. **b1**–**b2** From ref. ^53^. Reproduced by permission of Springer Nature: Light: Science & Applications. **c1**–**c2** Reproduced from ref. ^54^ with permission from ACS Publications. **d** From ref. ^51^. Copyright 2015, Wiley-VCH. **e** Reproduced from ref. ^178^. Copyright 2016, Wiley-VCH

The optical sensors transform environmental stimuli into the modulated light signal, which is widely implemented for the ever-growing demand of Internet of Things^88,244^. Generally, the 2D-material-integrated optical fiber sensors hold high sensing performance considering the fact that their optical responses are easily modulated by the external stimuli^87,245–247^. In the last few years, researchers have developed various hybrid-fiber schemes for physical^52,184,248,249^ and chemical sensing^24,88,244,250–252^. In particular, graphene and related 2D materials are appealing platform for chemical molecules sensing since they have ultimate surface-to-volume ratio, large adsorption capacity and ultrafast carrier mobility^88,244,253^. In principle, the adsorption of molecules changes the permittivity of the 2D materials, which in-turn modulates the parameters of coupling light source, i.e. amplitude, phase, polarization and wavelength^24,88^. The guided evanescent wave in either MF or DSF waveguides^88,251,252,254^ with the fiber grating^48,255,256^, interferometer^257,258^ and microresonator^52,259^ structures are widely studied to increase the sensor sensitivity and reduce the detection limit. For example, Wu et al.^48^ reported a graphene-coated MF Bragg-grating for sensitive gas sensing as shown in Fig. 7a1–a2, and the obtained sensitivities are 4 pm/ppm and 2 pm/ppm for ammonia and xylene gas, respectively. Hao et al.^260^ demonstrated graphene-based ammonia sensor using an in-fiber Mach-Zehnder interferometer with a sensitivity of ~3 pm/ppm. Note that most of the work are based on graphene, which often suffer from the cross-talk and limited selectivity problems, it is promising to explore other 2D materials, heterostructures or surface functionalizations to achieve high-selectivity label-free sensors^244,261–263^. Besides the aforementioned passive sensing, Cao et al. recently demonstrated graphene-enabled fluorescent resonance energy transfer in fiber-microfluidic resonator for ultrasensitive and selective biochemical detection, as shown in Fig. 7b1–b2^53,264^. They achieved individual-molecule sensitivity for dopamine, nicotine and single-strand DNA detection through dual amplifications from optical pump and electrical locked-in detection^53^. An et al.^54^ achieved individual gas molecule detection employing electrically tunable four-wave-mixing effects in graphene bipolar-junction-transistor heterogeneous DSF, as shown in Fig. 7c1–c2.

The atomic-layer thickness of 2D materials with supreme mechanical properties enables high-performance MEMS for fiber sensing applications^50,138^. Figure 7e shows the typical structure of a fiber-integrated MoS_2_-MEMS sensors, in which the free-standing MoS_2_ diaphragm and fiber endface form a Fabry-Perot interferometer^178^. The external stimuli deform the MoS_2_ membrane and change the cavity length, which shifts the optical interference spectra. The relation between the deflection of the diaphragm and external pressure could be modeled as^178^:

3 $$P = \frac{{A\sigma _0t{\Delta}L}}{{r^2}} + \frac{{BEt{\Delta}L^3}}{{(1 - \upsilon )r^4}}$$

where *A* and *B* are dimensionless coefficients, *P* is the applied pressure, σ_0_ is the pre-stress, *r* and *t* are the radius and thickness of a circular diaphragm, Δ*L* is the center deflection of diaphragm exposed to the pressure, *E* and *υ* are materials Young’s modulus and Poisson’s ratio respectively. Ma et al.^50^ first reported a miniature fiber-tip pressure sensor using a few-layer graphene as a diaphragm, and they observed a spectra sensitivity over 39.4 nm/kPa. Later, higher pressure sensitivity is achieved in MoS_2_ diaphragm sensors, as anticipated by their reduced Young’s modulus and improved film quality (Fig. 7e)^178^. Zheng et al.^51^ demonstrated ultrasensitive (2.2 × 10^5^ nm/A^2^) and fast-response (~0.25 s) electrical current sensor by depositing both gold electrodes and graphene membrane on an etched fiber tip, as shown in Fig. 7d. The highly efficient and localized ohmic-heating, and high thermal conductivity in graphene film synergistically contribute to the high-performance sensors. Besides the quasi-static deformations, the intrinsic nanomechanical resonators by clamped 2D materials also allow the development of vibrational fiber-optic sensors for robust force, mass and pressure measurements^265,266^. Note that both the resonating frequency and quality factor of 2D materials in the fiber platform are far less than the on-chip devices^267,268^, and further research are needed to optimize the 2D materials geometry and manipulate the pre-stress in fiber devices.

### Nonlinear optics

**Fig. 8:**
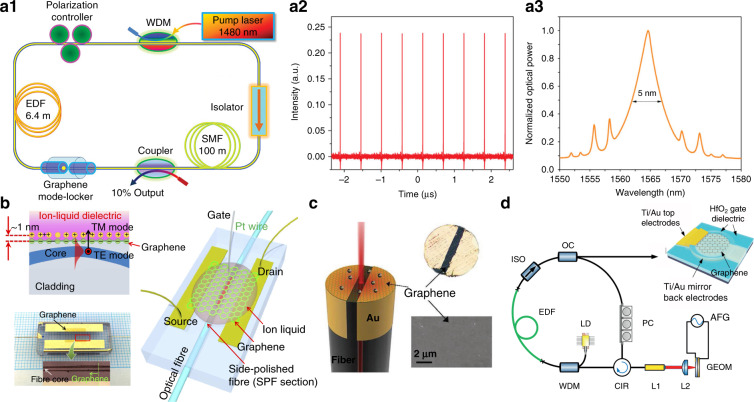
Tunable graphene-based saturable absorber for mode-locked fiber laser. Laser configuration constituting a ring cavity (**a1**), typical laser output pulse train (**a2**), output pulse spectra centered at 1567 nm with solitonic sidebands (**a3**)^44^. Tunable graphene saturable absorbers by electro-static gating (**b**)^96^ and electric current effect (**c**)^94^. **d** Actively mode-locked laser with a graphene-based EOM^272^. **a1**–**a3** From ref. ^44^. Copyright 2009, Wiley-VCH. **b** From ref. ^96^. Reprinted with permission from Springer Nature: Nature Communications. **c** Reproduced from ref. ^94^ with permission by Wiley-VCH, Copyright 2017. **d** From ref. ^272^ Copyright 2018, Wiley-VCH

Nonlinear optics is the study of the phenomena that optical response of materials are modified by the light field, and it has found broad applications in novel light source generating, signal processing and optical imaging. The state of art 2D materials have enabled many scientific advances in nonlinear effects, such as saturable absorption, Kerr effect, harmonic generation and parametric oscillation^93,189^. In particular, the saturable absorbers (SA) that realize high (low) transmittance of high (low) power density beam, are comprehensively studied in 2D materials for pulse laser generation, of which the laser wavelength spans from visible to the mid-infrared and the pulse width ranges from microsecond to sub-picosecond^92,93,269–271^. For example, Bao and Sun et al.^44,45^ pioneered the study of the graphene mode-locked ultrafast laser by simply depositing graphene on a fiber endface, as shown in Fig. 8a1–a3. Compared with conventional semiconductor SA mirrors (SESAMs) and nanotubes, graphene SA is found to have an intrinsic wideband operation^175^. Moreover, the linear Dirac-cone electronic bandstructure allows the tunable saturable absorption by either electric gating or thermal effect^95^, thus the pulsed laser state can be actively controlled. Lee et al.^96^ first reported electro-static gating in graphene-DSF devices (Fig. 8b), and they realized electrically tunable fiber laser at various operational regimes. Later, Li et al.^94^ demonstrated state-variable fiber laser by engineering the Fermi-Dirac distribution of graphene based on an electric heating method (Fig. 8c). Recently, Bogusławski et al.^272^ adapted graphene-based EOM into a fiber cavity (Fig. 8d), and obtained electrically controlled repetition rate of generated pulses.

**Fig. 9:**
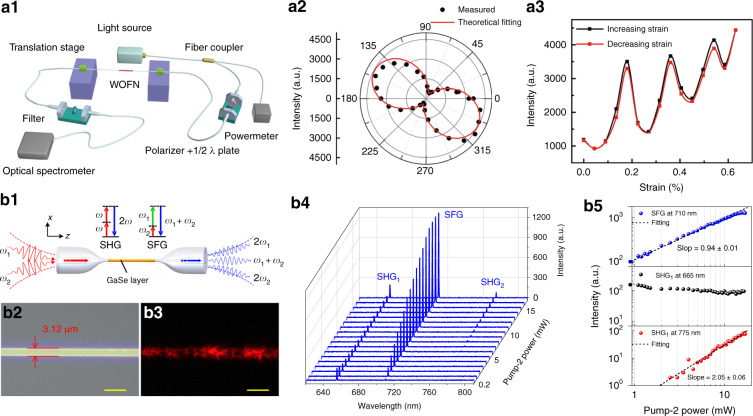
Second-order nonlinear optics in hybrid microfiber-2D-materials structure. **a** Tunable and enhanced second harmonic generation (SHG) in hybrid WS_2_-MF waveguide^182^. Experimental setup for SHG measurement (**a1**), output SHG depending on pump-light polarization (**a2**) and output SHG intensity relating to loaded strain (**a3**)^182^. **b** High-efficiency SHG and sum-frequency generation (SFG) in GaSe nanoflakes functionalized MF^274^. Schematic of operations of SHG and SFG from GaSe-integrated MF (**b1**), Optical microscopic images of samples (**b2**, **b3**), spectral evolution of SHG and SFG with variation of the Pump-2 light (**b4**) and Log-log plots of the power dependences of SFG (top), SHG_1_ (middle) and SHG_2_ (bottom) by varying the incident power of Pump-2 (**b5**)^274^. (**a1**–**a3**) and (**b1**–**b5**) from ref. ^182,274^ with permission by Springer Nature: Light: Science & Applications

The integrations of 2D materials in fiber waveguide structures^54,56,180,182,273,274^ are effective methods to enhance and manipulate the nonlinear optical interactions. Wu et al.^186,275^ reported cascaded four-wave-mixing with graphene-coated-MF structure because of graphene’s ultrahigh third-order nonlinearity. Chen et al.^182^ systematically studied the anisotropic response of SHG in hybrid WS_2_-MF as shown in Fig. 9a1–a2; furthermore, they demonstrated dynamic control of SHG by strain gauge (Fig. 9a3). Jiang et al.^274^ reported high-efficiency second-order nonlinear processes (SHG and sum frequency generation) in an MF assisted by few-layer GaSe as shown in Fig. 9b1–b5. Fundamentally, 2D-materials enhanced optical nonlinearity is limited by the trade-off between absorption and interaction length. The defects absorption/scattering introduced during 2D materials transfer processes are always serious issues in current optical devices. Recently, Zuo et al.^196^ reported high crystalline as-grown MoS_2_ in 25-cm long HCF, and they observed that both SHG and third-harmonic generation (THG) were enhanced by ~ 300 times compared with monolayer MoS_2_/silica. This work will inspire development of clean 2D-materials-fiber devices with great potential of mass production and stimulate versatile nonlinear applications. Besides using their intrinsically high optical nonlinearity, the electrically tunable nonlinear response^97,98,132^ and plasmonic-enhanced nonlinearity in nanostructured 2D materials^276,277^ are promising directions for reconfigurable nonlinear fiber devices, which is difficult to achieve with conventional bulk materials.

### Challenges and opportunities

In the past years, the silica optical fibers merged with 2D materials have stimulated rapid progresses for in-line manipulating light beams in respect of polarization, phase, intensity and frequency, which shed light on the integrated all-fiber photonic and optoelectronic systems. Note that most of the applications remain at proof-of-concept or prototype stages, and many key challenges, such as batch reproduction of devices and reliable packaging are still waiting to be addressed towards the ultimate practical applications. With advanced fiber manufacturing, there are sophisticated techniques to fabricate various bulk fiber structures such as DSF, PCF, HCF and cleaved fiber-endface. With respect to materials production, different forms of 2D materials from the solution-processed nanosheets to the CVD-grown large-size single crystals are already available^68,278,279^. Nevertheless, the conventional mechanical transfer of 2D materials to fiber structures are time consuming and not scalable, and the unintentional doping and structural defects are easily introduced to hybrid fiber devices, which will significantly influence their optoelectronic performance^46,56,182,185,192,235,280^. The recently developed functional inks and prints of 2D materials^281^ are gaining momentum for mass production with a high speed, low cost and moderate resolution (< 100 μm), and is potential for fabricating fiber devices that does not require crystallined 2D materials^88,252,282^. Using a direct CVD growth method to produce polycrystalline structure of 2D materials (graphene and MoS_2_) in PCF/HCF has also been achieved in 2019^195,196^, which may provide the ultimate solution to most of the hybrid fiber devices. Device packaging is another vital issue to realize long-term and stable operation of 2D materials especially for air unstable materials, such as BP, MoTe_2_ and Bi_2_Se_3_^145,283,284^. The excellent electrical insulation, high thermal stability and chemical inertness render hexagonal boron nitride as one of the most important candidates for passivation and protection layer^285^.

As for the future development of hybrid fiber devices, the novel materials and advanced structures are two important ingredients for photonic and optoelectronic integration. Beyond the conventional graphene, BP and TMDCs, lots of other layered and non-layered 2D materials are discovered with diverging properties^70,71,233,286^, and their Van der Waals heterostructures further reveal unusual physics and properties^66,67,287,288^. In particular, the 2D magnets such as CrI_3_ and Fe_3_GeTe_2_ with magneto-optical Kerr effect show great potential for optical non-reciprocal fiber-devices at room temperature^289,290^. The twisted 2D materials with Moire pattern in graphene and TMDCs demonstrate exotic optical and electronic properties, which is intriguing to extend the optical spectra of photodetection and light emitting^288,291^ in fibers. In the scope of photonic structures, the interaction of confined chiral field in MF^191^ and valley polarization in TMDCs^117–119^, such as MoS_2_ and WS_2_, may promise novel all-fiber optical routers^122^. The BP of highly anisotropic linear and nonlinear optical response interacts the vector field in optical fiber modes should create extraordinary polarimetric fiber devices. On the other, the adaptation of external nanophotonic structures in DSF and fiber endface combining with 2D materials will achieve ultimate all-fiber light-matter interactions and light beam manipulation^177^. Beyond the classical-optic applications, the subwavelength MF is an efficient interface for coherent transfer of quantum states between atomic and photonic qubits, due to its strong transverse confinement of the guided field and the long interaction length along the propagation^212^. The emergence of 2D materials based SPEs in MF platform may provide an alternative fiber-integrated source and open promising new avenues for quantum photonics.
